# Electrical Properties and Kinetics of Electrode Reactions

**DOI:** 10.6028/jres.065A.032

**Published:** 1961-08-01

**Authors:** Ralph J. Brodd

## Abstract

A common basis for investigations of the properties of electrode reactions is provided. The basic equations of electrostatics and electrodynamics and the assumption that electrode reactions are relaxation processes, are used to develop the equations for the electrical behavior of electrode systems. Thus electrode reaction processes are characterized as two states separated by an energy barrier. The application of static and alternating fields to electrode systems is interpreted in terms of the kinetic parameters of the electrode reactions. The equations for impedance are applied to silver and cadium electrode systems reported in the literature. The agreement of experiment and theoretical expectation is excellent. The equations are also applied to the interpretation of the impedance of LeClanché cells. The kinetic analysis of a simple unimolecular reaction is used to illustrate the kinetic interpretation of experimental information. This simple analysis may be extended to more complex reactions.

## 1. Introduction

The experimental investigation of electrode reaction mechanisms has followed four schemes of attack: (a) the determination of the steady state electrode potential as a function of current, (b) the voltage-step or potentiostatic method where the current density is measured as a function of time at constant electrode potential, (c) the current-step or galvanostatic method where the electrode potential is measured as a function of time at constant current density, and (d) the measurement of the impedance of the electrode system as a function of the frequency of an applied alternating field. The analysis of method (a) has been summarized in great detail by Bockris [1].^1^ The theoretical basis for analysis of methods (b), (c), and (d), however, is not complete and in many cases the fundamental relationship between these methods is not recognized.

Method (d), in spite of its wide application, has suffered in the past from several disadvantages. Contamination problems especially on solid electrodes are intensified as adsorption and desorption of solution impurities give rise to an impedance in parallel with the electrode impedance. Corrosion reactions at metal electrodes give rise to impedances which also contribute to the overall electrode impedance. The measurement of the high capacitance and low resistance of electrode processes also presents some experimental difficulties.

Over the past sixty years the accepted theory for the prediction of the electrical properties of electrode systems has been based primarily on the work of Warburg [2]. Other theories which postulate either that the electrode capacitance is a power function of the frequency [3] or that a special circuit element of constant phase angle *θ* is present [4] do not conform with experimental evidence [5]. Warburg assumed that diffusion of the reacting species to the electrode surface was the cause of electrode polarization. His theory predicted that the phase angle was 45° and that the series resistance and capacitance of the electrode varied as *ω*^−1/2^ where *ω*=2*πf* with *f* the frequency of the alternating field. The mathematics of the original theory of Warburg has been improved by many people. The most significant improvement in recent years removed the restriction of a constant phase angle [6]. However, even with this restriction removed there are serious discrepancies between the theory and experiment.

These developments all predict a linear dependence of the resistance and reactance with *ω*^−1/2^. While this relation is obeyed in many instances, frequent references are found to “anomalous” dispersions and absorptions. The cause of the disagreement between experiment and theory has been suggested to be surface roughness, adsorption and desorption of solution impurities or the presence of a corrosion process. The point of view in this paper is that the basis of earlier theories is unsatisfactory. A new representation for the consideration of electrode processes which includes the earlier theories will be set forth in this paper.

Since electrode processes may be described as involving two equilibrium states separated by an energy barrier [7], it is convenient to treat electrode reactions as relaxation processes. The controlling relaxation process may be either a charge transfer reaction (Ag=Ag^+^+*e*), a chemical reaction simultaneous with, preceding, or succeeding, the charge transfer reaction, or a diffusional process of one or more of the participants in the reactions at the electrode-solution interface. Data from the existing literature will be used as a test for the treatment of electrode processes given below. It is the main purpose of this paper to demonstrate the application of relaxation theory and methods to impedance measurements of the electrode-solution interface.

## 2. General Relaxation Theory

### 2.1. Electrical Theory

Recently there has been increased interest in the electrostatic approach to electrochemical kinetics [8]. In particular, an attempt to relate atomic and electronic polarizations to the calculation of oxidation-reduction reaction rates has been made by Marcus [9] with some success. Relaxation theory has been applied to evaluate the reaction rate constants of ionization reactions in aqueous solutions with good success [10].

**Figure 1:**
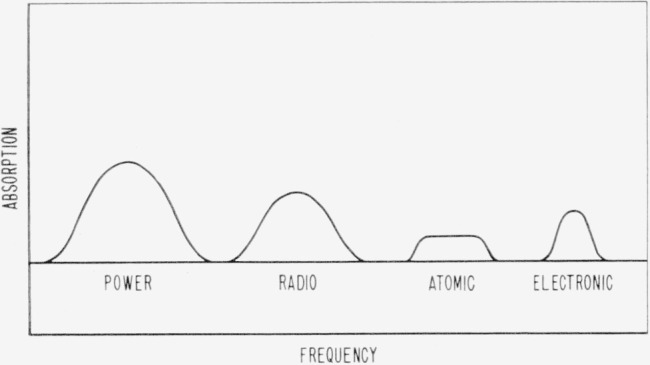
Energy absorption as a function of frequency for a hypothetical system.

As a result of these successes in applying relaxation theory to electrolytic systems it is appropriate to attempt to apply the same theoretical consideration to the electrode-solution interface. Both the Debye theory [11] which treats polarization arising from the orientation of permanent dipoles and the Wagner-Maxwell theory [12] which treats polarization arising from the accumulation of charges at the interface in heterogeneous dielectrics, lead to energy absorption as characterized in figure 1 for a hypothetical case. Electrode processes also lead to energy adsorption at the electrode-solution interface, often in the power frequency range.

While the derivation of the equations for relaxation processes are well known and are available elsewhere [12], a brief account of the development is in order.

We will define the field quantity **E** by

E=−∇V(v/m), (1)

where *V* is electric potential. The vector polarization, **P**, is defined by

$$P=D-\epsilon_{0}E\;(\text{coulombs}/m^{2}),$$ (2)

where *ϵ*_0_ is the permittivity of a vacuum and **D** is the displacement. The relationship between **E** and **D** is given by

$$D=\epsilon E\;(\text{coulombs}/m^{2}),$$ (3)

where *ϵ* is the permittivity.

When the electrode-solution interface is subjected to an alternating field, two possibilities arise which depend on the frequency of the field, the temperature, and the nature of the electrode-solution interface. In the first case there is no measurable phase difference between **D** and **E** and the polarization is in phase with the alternating field. In the other case there is a measurable phase difference between **D** and **E**. A phase difference between **D** and **E** can be due to any of three factors: d-c conductivity, relaxation effects, and resonance effects.

We will examine now in more detail relaxation effects as they affect the energy absorption at the electrode solution interface. A simple model that may be used for the description of this effect is that of a process in which the energy absorption is characterized by a relaxation time. If a constant electric field is applied at the electrode-solution interface we assume that a polarization will result from a disturbance of the equilibrium distribution of the participants in the electrode reactions at the electrode-solution interface. We will also assume as in other relaxation phenomena that the time rate of change $\frac{dP}{dt}$ of **P** is proportional to the difference between the final value, *P_s_*, and the actual value *P* [11, 12]:

$$\frac{dP}{dt}=\frac{1}{\tau }\;(P_{s}-P)\;(\text{coulombs}/m^{2}/\sec),$$ (4)

where *τ* is a constant with the dimensions of time. Since *τ* is a measure of the time lag, it is called the relaxation time. Integrating eq (4) using the condition *P_t=_*_0_*=P*_∞_ (i.e., *P*_∞_ is the instantaneous contribution to the polarization) we obtain

$$P=P_{\infty }+(P_{s}-P_{\infty })\;(1-e^{-t/\tau })\;(\text{coulombs}/m^{2}).$$ (5)

In the opposite condition where a static field is suddenly taken away we have

$$\frac{dP}{dt}=-\frac{1}{\tau }P\;(\text{coulombs}/m^{2}/\sec).$$ (6)

Here we have *P_t=_*_0_*=P_s_*−*P_∞_.* Integration gives:

$$P=(P_{s}-P_{\infty })e^{-t/\tau }(\text{coulombs}/m^{2}).$$ (7)

A description of relaxation effects by equations similar to eqs (5) and (7) was first given by Pellat [13]. The subscripts ∞ and *s* refer to the values of the quantities at times much less or much greater than *τ*.

For alternating field when there is a phase difference between **D** and **E**, it is useful to express *D* and *E* as complex numbers,

$$E=E_{0}e^{+j\omega t}\;(v/m)$$ (8)

$$D=D_{0}e^{+j(\omega t-\delta )}\;(\text{coulombs}/m^{2})$$ (9)

where *j* is $\sqrt{-1}$, *ω*=2π × the frequency *f* of the alternating field, *t* is the time, and *δ* is the phase angle between **D** and **E**.

When *δ* is independent of **E** we can write

$$D=\epsilon *E\;(\text{coulombs}/m^{2}).$$ (10)

where ϵ* is the complex permittivity. Here we will be most interested in the impedance, *Z* of the electrode-solution interface,

$$Z=R-jX_{c}\;(\text{ohms}),$$ (11)

in which *R* is the resistance and *X_c_* is the capacitive reactance.

In relaxation theory it is generally assumed that eq (4) is valid for alternating as well as static fields, and hence it can be shown that^2^

$$Z=R_{\infty }+\frac{R_{s}-R_{\infty }}{1+j\omega \tau }\;(\text{ohms}).$$ (12)

As a result

$$R=R_{\infty }+\frac{R_{s}-R_{\infty }}{1+\omega^{2}\tau^{2}}\;(\text{ohms}),$$ (13)

and

$$X_{c}=(R_{s}-R_{\infty })\frac{\omega \tau }{1+\omega^{2}\tau^{2}}\;(\text{ohms}).$$ (14)

**Figure 2:**
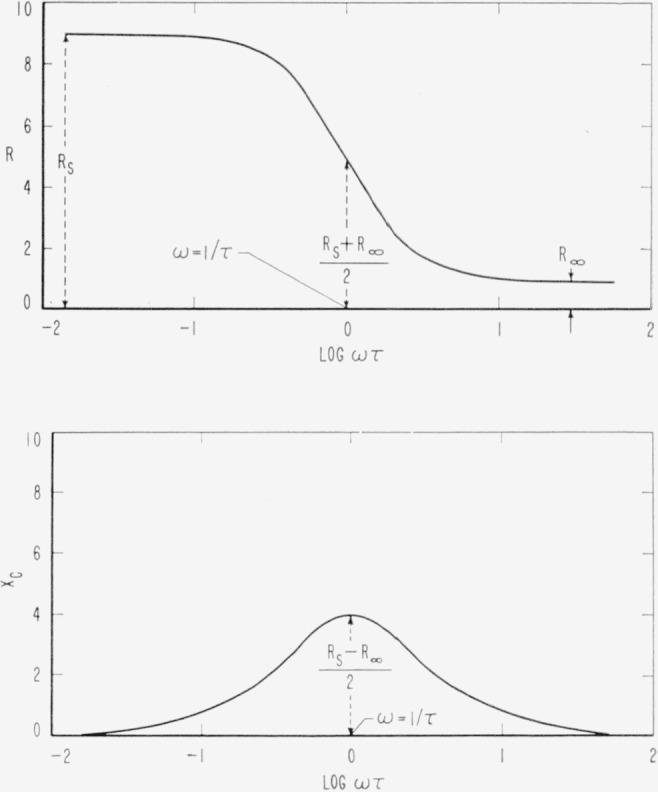
The dependence of R on frequency according to eq (13) assuming R_s_=9, R_∞_ = 1 and τ=10^−3^ sec.The dependence of X_c_ on frequency according to eq (14), assuming R_s_=9, R_∞_ = 1 and *τ* = 10^−3^ sec.

**Figure 3:**
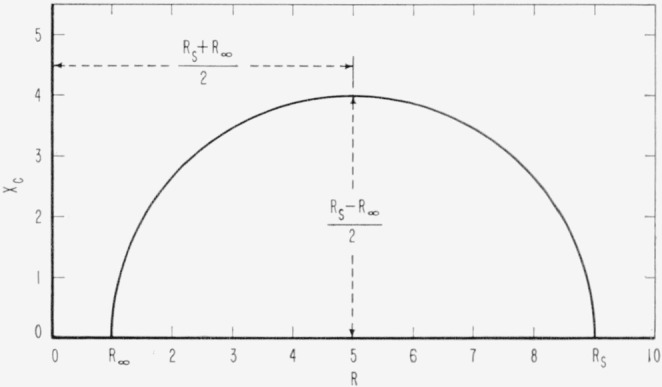
Relationship between R and X_c_ according to eq (18), for R_s_, R_∞_ and *τ* given in figure 2.

By plotting the left hand side of eqs (13) and (14) against log *ω* we obtain the plots shown in figures 2 and 3. The maximum in the *X_c_*−log *ω* plot is reached when log *ωτ*=0. Thus

$$\omega_{\max}=\frac{1}{\tau }\;(\sec^{-1}).$$ (15)

We have

$$R_{\left(\max\right)}=\frac{R_{s}+R_{\infty }}{2}\;(\text{ohms}),$$ (16)

$$X_{c(\max)}=\frac{R_{s}-R_{\infty }}{2}\;(\text{ohms}).$$ (17)

The use of a logarithmic scale has the advantage that the curves in figures 2a and b are symmetrical about *ωτ*=1.

Another method of representing the curves is to construct an Argand diagram or complex plane locus in which the imaginary part *X_c_* of the impedance is plotted against the real part *R*, each point corresponding to one frequency (13). From eqs (13) and (14) we obtain

$${\left[R-\frac{R_{s}+R_{\infty }}{2}\right]}^{2}+X_{c}^{2}={\left[\frac{R_{s}-R_{\infty }}{2}\right]}^{2}.$$ (18)

By plotting *R* versus *X_c_* a semicircle must be obtained with a radius (*R_s_*−*R*_∞_)/2, its center on the abscissa at a distance (*R_s_+R*_∞_)/2 from the origin. The semicircle in figure 3 corresponds to values taken from figure 2. For given values of *R_s_* and *R*_∞_ the *R, X_c_* curve is completely defined provided the equations are valid. The frequency range in which the absorption occurs has no influence on the *R, X_c_* curve. Thus, this method of representation is independent of the relaxation time, and reveals the inherent simplicity of the relaxation process.

An experimental quantity of interest is tan *θ*where *θ*=90°−*δ.* From eqs (13) and (14) we find

$$-\tan\;\theta =\frac{X_{c}}{R}=\frac{(R_{s}-R_{\infty })\omega \tau }{R_{s}+R_{\infty }\omega^{2}\tau^{2}}.$$ (19)

Taking (∂ tan *θ*/∂*ω*) = 0 we find the maximum value of tan *θ* is obtained at the frequency

$$\omega_{\max}^{\prime }=\frac{1}{\tau }\sqrt{\frac{R_{s}}{R_{\infty }}}(\sec^{-1})$$ (20)

$$-\tan\;\theta_{\left(\max\right)}=\frac{R_{s}-R_{\infty }}{2\sqrt{R_{s}R_{\infty }}}.$$ (21)

**Figure 4:**
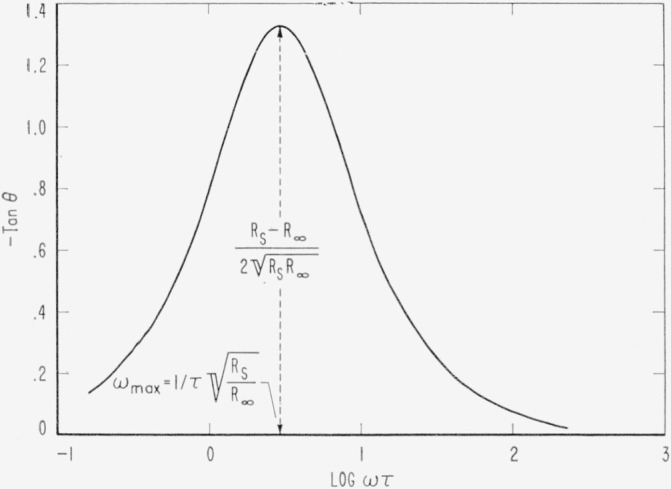
The dependence of tan θ on frequency according to eq (19) for R_s_, R_∞_ and *τ* given in figure 2.

In figure 4 the dependence of tan *θ* on log *ω* is shown for the values of *R_s_, R*_∞_ and *τ* used in figures 2 and 3.

**Figure 5:**
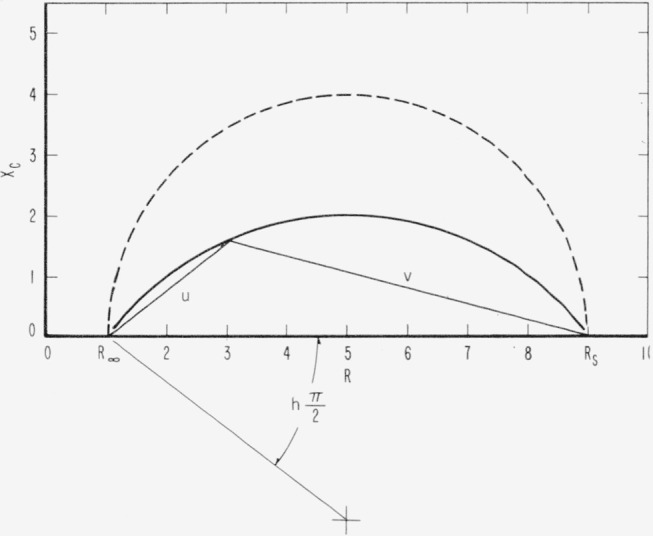
Relationship between R and X_c_ according to eq (26), defining the parameter, h for R_s_, R_∞_ and *τ* given in figure 3. The curve for *h*=0 is drawn in with dashed lines.

In some cases [14] *τ* may have a distribution of values about a most probable value. The fact that *τ* may have a distribution of values in no way invalidates the equations or graphical representations given previously. Rather it throws eq (12) into a more general form:

$$Z=R_{\infty }+\frac{R_{s}-R_{\infty }}{1+j{\left(\omega \tau \right)}^{1-h}}\;(\text{ohms})$$ (22)

where *h* is identified with the angle defined in figure 5. When *h*=0 eq (22) is reduced to eq (12). The distribution function for the case where *h*=0 is closely approximated by a Boltzman distribution function for τ.

A point is chosen on the *R, X_c_* curve in figure 5 corresponding to a measurement at a certain frequency *ω*_1_. The distances *u* and *v* from this point to the intersection points *R*_∞_ and *R_s_* of the curve with the abscissa respectively are determined as shown in figure 5. The equation

$$u/v={\left(\omega_{1}/\omega_{\max}\right)}^{1-h}$$ (23)

may be used to evaluate *ω*_max_ if *u, v, ω*_1_, and *h* are known. To determine *h* a plot of the quantity log (*u/v*) versus log *ω* is constructed. The result must be a straight line with the slope (1−*h*) in order that eq (22) be applicable.

In this discussion only one process was assumed to be occurring. Wherever more than one relaxation process is occurring simultaneously, the total polarization is assumed to be the sum of the different polarization contributions. As a result eq (5) is written in the form

$$P_{t}=P_{\infty }+\sum_{r}{\;(P_{s}-P_{\infty })}_{r}(1-e^{-t/\tau_{r}})\;(\text{coulombs}/m^{2})$$ (24)

and eq (12) is written as

$$Z=R_{\infty }+\sum_{r}\frac{{\left(R_{s}-R_{\infty }\right)}_{r}}{1+(j\omega \tau_{r})}\;(\text{ohms})$$ (25)

with

$$R=R_{\infty }+\sum_{r}\frac{R_{a}}{1+\omega^{2}\tau_{r}^{2}}\;(\text{ohms})$$ (26)

and

$$X_{c}=\sum_{r}\frac{R_{a}\omega \tau_{r}}{1+\omega^{2}\tau_{r}^{2}}\;(\text{ohms})$$ (27)

where

$$R_{a}={\left(R_{s}-R_{\infty }\right)}_{r}$$ (28)

is the contribution to the resistance by the *r*-th relaxation process. If eq (25) does not apply, a more powerful means of combining the impedance of the various processes must be found.

### 2.2. Kinetic Theory

**Figure 6:**
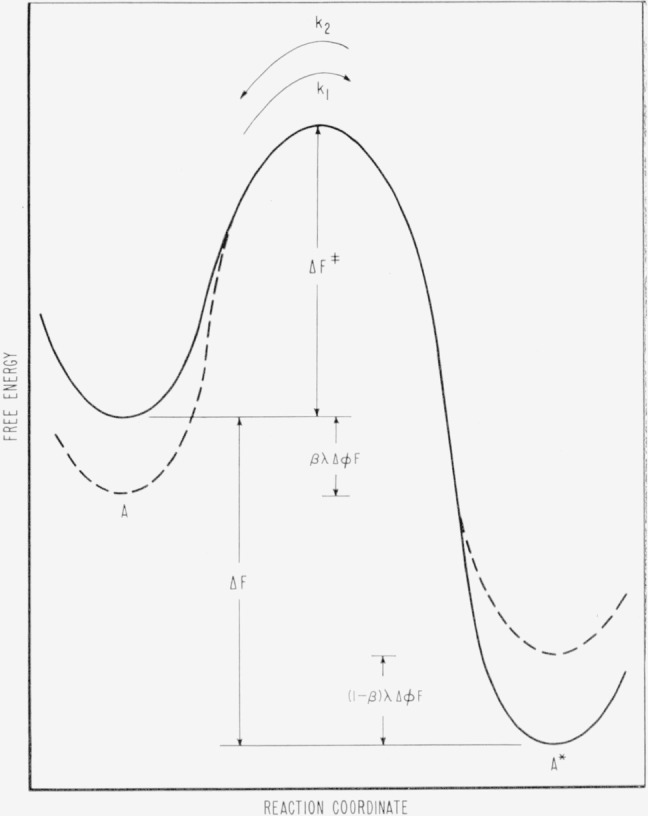
Details of the free energy of activation barrier for a simple unimolecular process.

In section 2.1, it was assumed that polarization is the result of a disturbance of the equilibrium distribution of the participants in the electrode reaction. An alternate and sometimes more illuminating approach to the properties of the electrodesolution interface is based on the kinetic behavior of the various processes at the electrode-solution interface. In order to discuss the kinetics of an electrode process, it is necessary first to formulate a reaction mechanism. If the electrode process is a simple unimolecular reaction 3 given by

$$A\overset{k_{1}}{\underset{k_{2}}{⇌}}A*$$ (29)

the reaction may be depicted in terms of free energy and reaction coordinates as shown in figure 6 [15].

The reaction rate constants are related to the free energy of activation and the field strength by

$$k_{1}=G_{0}\;\exp-\left[\frac{\Delta F^{‡}+\lambda \beta \Delta \phi F}{RT}\right]\;(\sec^{-1})$$ (30)

and

$$k_{2}=G_{0^{\prime }}\;\exp-\left[\frac{(\Delta F^{‡}+\Delta F)-\lambda (1-\beta )\Delta \phi F}{RT}\right]\;(\sec^{-1})$$ (31)

where *G*_0_ and *G*_0′_ are constants for a given system, Δ*F*^‡^ is the free energy of activation, Δ*F* is the free energy change for the process, λ is the number of charges transferred in the process, *R* is the gas constant, *T* is the temperature in degrees Kelvin, *F* is Faraday’s constant, *β* is the symmetry factor, usually assumed to be ½ [17] and Δ*ϕ* is the difference in the inner potentials of the solution and electrode. We may write the kinetic equations for the reaction directly.

$$dN_{1}/dt=k_{2}N_{2}-k_{1}N_{1}$$ (32)

$$dN_{2}/dt=-k_{2}N_{2}+k_{1}N_{1}$$ (33)

where *N*_1_ and *N*_2_ are the concentrations of *A* and A* respectively. If $N=\sum_{r}N_{r}$, it can be shown that solution of eqs (32) and (33) takes the form

$$N_{1}=N_{01}+C_{11}e^{-\alpha^{t}}$$ (34)

and

$$N_{2}=N_{02}+C_{12}e^{-\alpha^{t}}$$ (35)

where

$$\alpha =k_{1}+k_{2}\;(\sec^{-1})$$ (36)

and *N*_01_ and *N*_02_ are the equilibrium values of *N*_1_ and *N*_2_ respectively. Also we note that *C*_11_ = −*C*_12_*. C*_11_ is directly proportional to the relaxation time and conductance of the process and the potential drop at the electrode surface. Since *N*_2_−*N*_1_ is proportional to the polarization we note the exponential approach to equilibrium as required in relaxation theory. When a field is suddenly removed, it follows that

$$P=(P_{s}-P_{\infty })e^{-\alpha^{t}}\;(\text{coulombs}/m^{2}),$$ (37)

or

$$P=(P_{s}-P_{\infty })e^{-t/\tau }\;(\text{coulombs}/m^{2}),$$ (38)

which is identical with eq (7) and

$$\tau =1/(k_{1}+k_{2})\;(\sec).$$ (39)

Thus *τ* is related directly to the reaction rate constants for the process. The relaxation time *τ* depends on Δ*F* and Δ*F*^‡^ as well as Δ*ϕ*. As a result *τ* may be expected to be a function of temperature and electrode potential.

The resistance *R_s_*−*R*_∞_ eqs (12) and (25) is the resistance associated with the given relaxation process. This resistance will be the resistance of the electrode process, *R_a_*= (*R_s_*−*R*_∞_) ; *R_a_* may be substituted directly into the equation for exchange current density, *J*_0_,

$$J_{0}=(RT/\lambda F)\;(1/AR_{a})\;(\text{amp}/m^{2})$$ (40)

where [*RT*/λ*F*] is thermal potential expressed in volts and *A* is the area.

Either the Argand diagram or the log plots also may be used to evaluate the resistance of the electrode process, *R_a_*, and the relaxation time, τ. In figures 2b and 3, *R_a_*/2 is the maximum height of the curve. The frequency at the maximum is related to *τ* by eq (15). In figure 2a, *R_a_* is the height of the step and *τ* is found from the frequency at the midpoint of the step.

The equation from older theories (2, 6)

$$R-X_{c}=R_{a}$$ (41)

is not a reliable method for determining *R_a_* for an electrode process. From eqs (13) and (14) we see that the difference *R*−*X_c_* may vary from *R*_∞_ to *R_s_* depending on whether *ω* is far removed above or below *ω*_max_. Thus the use of eq (41) in the older theories is valid only at frequencies far below *ω*_max_ and if *R*_∞_ is zero.

At equilibrium

$$dN_{1}/dt=dN_{2}/dt=0.$$ (42)

The rate of the process at equilibrium is the exchange current. Therefore, we may write

$$J_{0}=k_{1}N_{01}=k_{2}N_{02}\;(\text{amp}/m^{2})$$ (43)

and

$$k_{1}/k_{2}=N_{02}/N_{01}.$$ (44)

From eqs (30) and (31) we can also write

$$k_{1}/k_{2}=\exp[(\Delta F-\lambda \Delta \phi F)/RT].$$ (45)

Unfortunately even if the free energy change is known, it is not possible at present to evaluate Δ*ϕ* for electrochemical processes. In certain circumstances a good approximation for Δ*ϕ* is given by

$$V_{e}-V_{zpc}=\Delta \phi \;(v)$$ (46)

where *V_e_*−*V_zpc_* is the electrode potential with respect to the electrode potential at zero point of charge [18]. The zero point of charge is very difficult to evaluate.

An alternate and possibly more fruitful approach to the ratio *k*_1_/*k*_2_ may be to determine the ratio *N*_02_/*N*_01_ as noted in eq (44). The concentrations of the elements of the process may be found by the analysis of the charge distributions. This type of calculation has been given for mercury electrode interfaces by Grahame [19]. At present, it is not possible to extend the analysis of charge distribution to other electrode interfaces except by inference as the zero point of charge for most metal electrodes cannot be accurately evaluated.

If one of the elements of the process is a metal, say *A*, the concentration of *A, N*_01_, may be taken as the number of surface atoms. Using eq (43) and the exchange current, *k*_1_ can be found immediately. Then substituting *k*_1_ and *τ* into eq (39)*k*_2_ can be evaluated. Likewise if *N*_02_, but not *N*_01_ may be determined, eqs (39) and (43) can be used to evaluate *k*_1_ and *k*_2_.

Once the ratio *k*_1_*/k*_2_ has been evaluated at one potential the rate constants for the process may be determined at any other potential by

$$k_{1}^{\prime }/k_{2}^{\prime }=(k_{1}/k_{2})\exp(-\Delta \phi \prime \lambda F)/RT$$ (47)

where Δ*ϕ*′ is the change in Δ*ϕ* from the old to the new electrode potential and $k_{1}^{\prime }/k_{2}^{\prime }$ is the ratio of rate constants at the new electrode potential.

## 3. Applications

**Figure 7:**
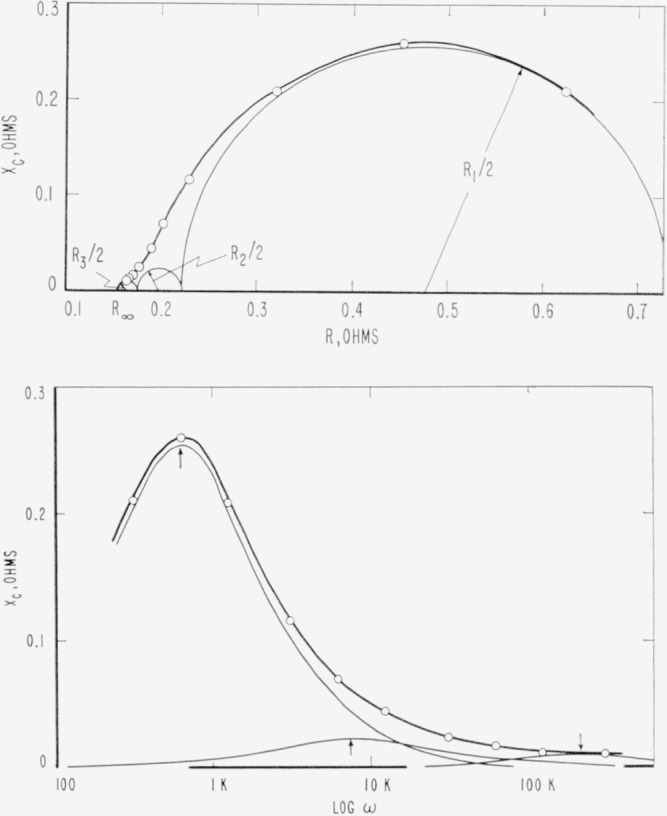
Argand diagram of the impedance of a LeClanché cell.The dependence of X_c_ on frequency for a LeClanché cell.

Since equations describing the kinetic and electrical properties of the electrode-solution interface are now available, the application of the various equations will be illustrated with data obtained from real electrode systems. The impedance of a D-size LeClanché cell constructed at NBS is shown in figure 7. The cell has a paste liner and a bobbin composition by weight of 8 parts African MnO_2_ ore, 1.24 parts ammonium chloride and 1 part acetylene black. The impedance of the cell was measured on a substitution type Wien bridge described by Vinal [20]. The values of the resistance, capacitance, and reactance of 4 cells are reported in table 1 for the frequency range 50 c/s to 50 kc/s at room temperature. From these data the values of *R_a_*, *ω_m_* and *R*_∞_ are given in table 2. Only data for cell 1 is shown in figure 7 for illustration. The introduction of three absorption regions was required to reproduce the impedance of LeClanché cells as a function of frequency. The three regions of energy absorption or dispersion were found in figure 7b by curve fitting using the theoretical behavior of the imaginary part of the impedance given by eq (27). These three curves are drawn in lightly in figure 7. It was necessary to introduce three relaxation processes in order to fit the experimental curve. The analysis of data by curve fitting is a necessity in the absence of other information on the location of the absorption regions.

Experiments using pairs of either zinc or manganese oxide electrodes established that the dispersion with *ω*_max_=600 c/s was associated with the zinc electrode while the other dispersions were associated with processes at the manganese oxide electrodes. Euler and Dehmelt [21] reached the same conclusions except they did not report the dispersion centered about *ω*_max_=180 kc/s. A summary of the results from other cells in the group manufactured at NBS is given in table 1. In all cases the sum of the theoretical curves for three absorption regions fit the experimental behavior of the impedance.

Figure 7 illustrates the usefulness of the various types of data representation. The Argand diagram (fig. 7a) immediately reveals the complete behavior of the impedance. The plot of log *ω* (fig. 7b) reveals the additivity of the imaingary part of the impedance as assumed in eq (27). Each type of data representation has its usefulness and applications.

**Figure 8:**
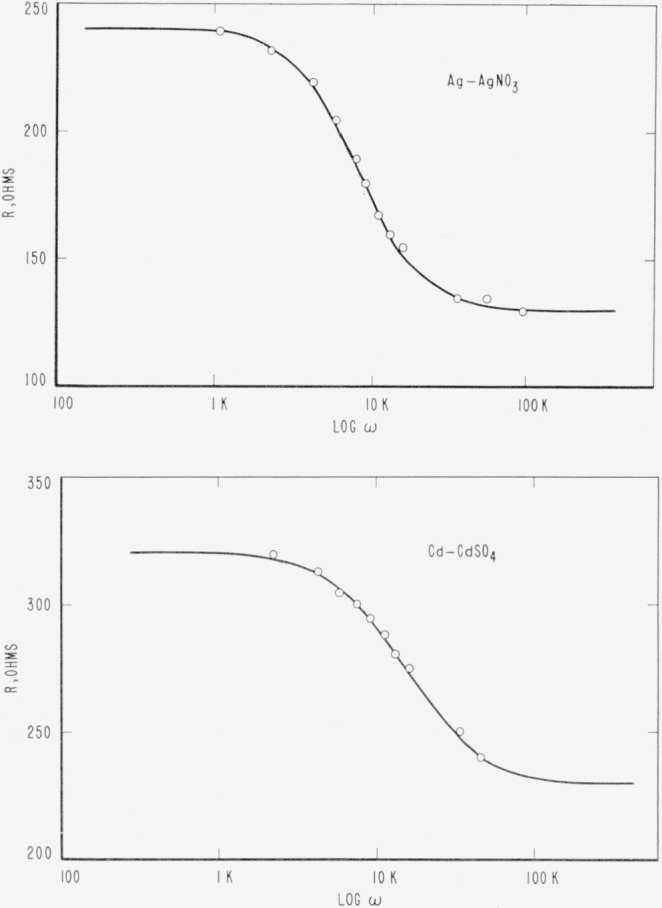
a. The dependence of resistance, R, on frequency for the silver-silver ion (10 g AgNO_3_ per 100 cm^2^ of solution) electrode system. The solid line was calculated using eq (25) and assuming that *R_s_*=242, *R*_∞_ = 130 and τ=1.23×10^−4^. The circles are the experimental data [23]. b. The dependence of resistance, R, on frequency for the cadmium ion (15 g CdSO_4_ per 100 cm^2^ of solution) electrode system. The solid line was calculated using eq (25) and assuming that *R_s_*=320, *R*_∞_=230 and τ=6.4×10^−5^. The circles are the experimental data [23].

The silver-silver ion and cadmium-cadmium ion electrode systems illustrate the use of eq (13) to represent the behavior of the resistive portion of the electrode impedance [22]. In figure 8 the electrode resistance versus log *ω* for both systems is shown. The results of the analysis of these graphs is given in table 3. The theoretical curves included for comparative purposes were calculated assuming that the values of *R_s_* and *R*_∞_ and *τ* given in the caption of the figure. The agreement of theoretical calculation and experiment is excellent.

To illustrate the calculations of *k*_1_ and *k*_2_ for unimolecular reactions the analysis of the silver-silver ion system in figure 8a will be given. Examination of figure 8a shows only one dispersion region. Therefore we need consider only one reaction. It is very unlikely that two reactions in the silver system would have the same relaxation time. We will assume that the process at the Ag electrode is:

$$\mathrm{Ag}\overset{k_{1}}{\underset{k_{2}}{⇌}}{\mathrm{Ag}}^{+}+e.$$ (48)

We will assume that the concentration of electrons is very large compared to the other concentrations and that the concentration of electrons remains constant. Thus, eq (48) may be assumed to be a unimolecular reaction. The concentration of silver atoms will be taken as the number of surface atoms, approximately 6.0×10^18^ atoms/m^2^, calculated from the silver metal lattice dimensions [23]. Using eq (43) and data from Table 3 with *J*_0_= 1.1 × 10^2^ amp/m^2^ or a particle flow of 6.9 × 10^20^ particles/m^2^/sec.

$$\begin{array}{l} 6.9\times 10^{20}=(6\times 10^{18})k_{1}\;(\text{particles}/m^{2}/\sec)\\ \;k_{1}=115\;\sec^{-1}. \end{array}$$

From eq (39) with *τ* =1/8100

$$\begin{array}{l} 8100=115+k_{2}\;(\sec^{-1})\\ \;k_{2}=7985\;(\sec^{-1}). \end{array}$$

From eq (44) the concentration of silver ions, *N*_2_, is

$$\begin{array}{l} 115/7985=N_{2}/(6.0\times 10^{18})\\ \;N_{2}=8.6_{4}\times 10^{16}\;(\text{ions}/m^{2}). \end{array}$$

Other investigations of the silver-silver ion system have reported the presence of a low frequency dispersion [24] associated with a diffusion process. This is not noticed in Banerji’s work, figure 8a, used here for illustration [22]. A similar analysis may be made for the cadmium-cadmium ion system.

## 4. Concluding Remarks

In this paper a point of view of the kinetics and electrical properties of electrode systems has been taken that differs from previous theories. The description of electrode reactions as relaxation processes, which includes charge-transfer, and chemical and diffusion processes, is very general in nature. Since diffusion processes may be classified as relaxation processes, the representation given in this paper will include previous theories. Equations for the prediction of the electrical behavior of electrode processes have been given. The representation given in this paper based on relaxation phenomena was applied to the electrical behavior of electrodes previously reported in the literature, namely, Ag, Ag^+^ and Cd, Cd^+^; conformity with theoretical predictions was obtained. The representation was also applied successfully to the electrical behavior of LeClanché cells. Only cursory observations on the galvanostatic and potentiostatic methods have been made and the problem of nonlinear combinations of electrode processes has not been considered. Also, resonance effects have not been treated in this paper.

## Figures and Tables

**Table 1:** Resistance, capacitance, and reactance of D-size LeClanché cells

Cell	*f*=*ω*/2*π*	50	100	200	500	1.0 *k*	2.0 *k*	5.0 *k*	10 *k*	20 *k*	50 *k*
											
1	R ohm	0.623	0.453	0.219	0.228	0.203	0.185	0.176	0.171	0.166	0.164
	C *μ* F	15100	6110	3815	2740	2262	1812	1320	946	648	298
	X ohm	0.211	0.261	0.209	0 116	0.070	0.044	0.024	0.017	0.012	0.011
2	R ohm	0.642	0.439	0.306	0.218	0.191	0.175	0.167	0.156	0.154	0.153
	C *μ* F	12100	5435	3568	2632	2153	1697	1241	929.8	652.0	260.2
	X ohm	0.263	0.294	0.223	0.121	0.074	0.047	0.026	0.017	0.012	0.009
3	R ohm	0.942	0. 519	0.378	0.250	0.210	0.186	0.171	0.163	0.160	0.161
	C *μ* F	9350	3962	2510	1829	1495	1211	931.0	926.9	534.4	297.7
	X ohm	0.431	0.402	0.317	0.174	0.106	0.066	0.034	0.022	0.015	0.011
4	R ohm	0.488	0.355	0.260	0.180	0.167	0.159	0.150	0.145	0.142	0.142
	C *μ* F	16700	7361	4843	3580	2893	2227	1580	1139	805.0	360.2
	X ohm	0.191	0.216	0.165	0.089	0.055	0.036	0.020	0.014	0.010	0.009

**Table 2:** Interpretation of data from table 1

Cell	*R*_∞_	*Ra*_1_	*ω*_max_	*Ra*_2_	*ω*_max_	*Ra*_3_	*ω*_max_
							
	*Ohm*	*Ohm*	*sec*^−1^	*Ohm*	*sec*^−1^	*Ohm*	*sec*^−1^
1	0.159	0.518	596	0.054	6.9 *k*	0.020	210 *k*
2	.151	.602	502	.058	6.3 *k*	.014	190 *k*
3	.160	.608	583	.060	8.2 *k*	.020	190 *k*
4	.136	.420	690	.050	3.1 *k*	.010	170 *k*

**Table 3:** Summary of data from figure 9

Electrode System	*R*_∞_	*Ra*^1^	*τ*	Electrode area	*I*_o_
					
Ag−Ag+	*Ohms*	*Ohms*	*sec*	*m*^2^×*10*^4^	*amp/m*^2^
[10 g AgNO_3_ per 100 cm^2^ water]	130	112	1.2_3_×10^−4^	0.04	1.1×10^2^
Cd−Cd++					
[15 g CdSO_4_ per 100 cm^2^ water]	230	90	6.4×10^−3^	.04	1.4×10^2^

1 The experimental cell was composed of two identical electrodes separated by a solution containing ions of the electrode material. The total resistance associated with the electrode reaction is the sum of the resistance contribution of each electrode. As a result the resistance used in calculation is *R_a_*/2.

## Footnotes

1 Figures in the brackets indicate the literature references at the end of this paper.

2 The subscripts ∞ and *s* refer to the value of the quantities at frequencies far above or far below 1/2*πτ* respectively. The definition *Z*=1/(*jωϵ**) was used to relate the complex permittivity to the impedance.

3 The analysis of more complex reactions will not be considered here. The reader is referred to the available literature for several treatments of complex reaction kinetics [16].
